# Peroxiredoxin 4 Involved in Spermatogenesis by Affecting Oxidative Stress and Ferroptosis

**DOI:** 10.1111/andr.70221

**Published:** 2026-03-27

**Authors:** Shuning Yuan, Nini Wei, Weiwei Ma, Mengting Hu, Bei Zhang, Li Gao, Chao Gao, Xiaoyu Yang, Yan Meng, Jiayin Liu, Yugui Cui

**Affiliations:** ^1^ State Key Laboratory of Reproductive Medicine and Offspring Health, Clinical Center of Reproductive Medicine The First Affiliated Hospital of Nanjing Medical University Nanjing China; ^2^ Reproductive Medicine Center Affiliated Maternity and Child Health Care Hospital of Nantong University Nantong Jiangsu China; ^3^ Nanjing Pukou People's Hospital, Liangjiang Hospital of Southeast University Nanjing China

**Keywords:** ferroptosis, male reproductive aging, oxidative stress, peroxiredoxin 4, spermatogenesis

## Abstract

**Background:**

The main functions of testes are sperm production and androgen secretion in testicular befitting microenvironment. Excessive level of the reactive oxygen species (ROS) from metabolism and cellular events can lead to oxidative stress (OS) and ferroptosis, which injure the functions of mitochondria and endoplasmic reticulum (ER) of testicular cells, leading to the impaired spermatogenesis and spermiogenesis, as well as insufficient androgen. Our previous studies have shown that peroxiredoxin 4 (Prdx4) is a vital ER‐located protector against endoplasmic reticulum stress (ERS) in ovarian granulosa cells. In this study, we explored whether Prdx4 played a beneficial role in testes, and the potential application value in male reproduction.

**Methods:**

Prdx4 knockout mice and Prdx4^−/y^ Leydig cells were established using CRISPR/Cas9 system, the natural adult (9 weeks) mice, aging (9 months) mice, and wild‐type MLTC‐1 cells were used as control. To further investigate the protective effect of Prdx4 in the adult mice, the testicular 43°C water‐bath was used to induce an OS model.

**Results:**

The male Prdx4^−/y^ mice aged over 9 months showed the age‐related pathology of seminiferous tubule, the increased OS and ERS, and the decreased fertility. The adult Prdx4^−/y^ mice under heat stress manifested increased levels of OS, including almost 1.5‐fold rise in 4‐hydroxynonenal (4‐HNE) and 8‐hydroxy‐2′‐ deoxyguanosine (8‐OHdG), higher ferroptosis, and poorer fertility. Prdx4^−/y^ Leydig cells showed a higher level of ferroptosis after hydrogen peroxide treatment. ACSL4 increased by 18.6% while COX2 increased by 13.5%, and the expressions of GPX4 and SLC7A11 decreased by 49.2% and 24.3%, respectively. Increased levels of ferroptosis in MLTC‐1 leads to mitochondria‐derived OS and apoptosis, which can be partially restored by deferoxamine and overexpressing SLC7A11.

**Conclusion:**

We concluded that Prdx4 as a protective factor plays a critical role in preserving spermatogenesis by alleviating testicular OS and ferroptosis, and that Prdx4 may be known as a potential target in mitigating the adverse effects of natural aging and OS injury.

## 1 Introduction

Two main functions of testes are androgen synthesis and sperm production. The androgen synthesized in testicular Leydig cells determines male development, sex character, and sexual function, while the insufficient synthesis of androgen leads to the disorders of male development and sexual function [1]. The late‐onset hypogonadism (LOH) is also due to the progressive decline of androgen synthesis in middle‐aged and old men [2]. On the other hand, the spermatozoa produced in seminiferous tubule determine male fertility. From a clinical perspective, the disorders of spermatogenesis and spermiogenesis lead to male infertility.

Spermatogenesis is also associated with multiple cell death mechanisms, among which apoptosis and autophagy are well‐known examples. In recent studies, ferroptosis—a newly identified mode of regulated cell death—has been found to participate in this biological process as well [3, 4]. As an iron‐dependent cell death pathway, ferroptosis is mainly defined by three key features: the buildup of iron within cells, lipid peroxidation, and mitochondrial damage [5]. It has been confirmed that ferroptosis contributes to the development of various organ lesions and degenerative disorders, including cancer, ischemic injury to organs, neurodegenerative conditions, hepatopulmonary fibrosis, and autoimmune diseases [6]. When Fe^2^^+^ undergoes the excessive oxidation with peroxide groups, the harmful substances produced during this reaction can disrupt the integrity and stability of cell membranes [7]. Since sperm cell membrane contains the high level of polyunsaturated fatty acids (PUFAs), they are particularly susceptible to being damaged by reactive oxygen species (ROS), which in turn triggers lipid peroxidation [8]. However, how exactly ferroptosis affects spermatogenesis and male reproductive capacity is still not fully understood.

The evidence shows that the importance of the intratesticular microenvironment cannot be overemphasized for the productions of androgen and spermatazoa, including the balance of oxidation and anti‐oxidation system. The excessive level of the ROS from metabolism and cellular events can lead to oxidative stress (OS) and endoplasmic reticulum (ER) stress, which injure the functions of mitochondria and testicular cells [9, 10]. Ferroptosis induced by OS and ER stress (ERS) accelerates cellular aging and cellular dysfunction. Besides the diminished androgen synthesis in Leydig cells, OS impairs spermatogenesis and spermiogenesis by damaging Sertoli cells, spermatogonial stem cells and spermatocytes, and the vitality of sperm cells, showing a decrease in sperm count and an increase in teratozoospermia index [11, 12]. Clinically, the adverse factors such as aging, obesity, microbial infections, inflammation, and external physical stressors such as radiation and occupational heat exposure, are associated with the elevated OS [13, 14, 15]. Meanwhile, both enzymatic and non‐enzymatic antioxidant systems exist in cells to mitigate OS damage, such as the classical antioxidants of superoxide dismutase (SOD) and glutathione peroxidase (GPX). These antioxidant systems can directly scavenge ROS, terminate free radical chain reactions, repair the damaged molecules, and can regulate the redox balance [16, 17]. Peroxiredoxins (Prdxs) are an important family of antioxidant enzymes, while Prdx4 is an arrestive member. The Prdx4 gene located on the X chromosome produces two isoforms: somatic cell‐type Prdx4 (Prdx4s) and testis‐specific Prdx4 (Prdx4t) [18, 19].

Notably, Prdx4 is an endogenous antioxidant anchored to ER, with high expression level in the reproductive system [20, 21, 22]. Research showed that Prdx4 protects mouse ovary from the oxidative damage induced by D‐galactose, suggesting its protective antioxidant role in mitigating age‐related OS [23]. Our previous study showed that Prdx4 serves as a vital protector against ERS in ovarian granulosa cells. Prdx4 is also a critical oxidative folding protein that assists in the proper folding of FSH receptors in the ER of granulosa cells [24]. In this study, we established a Prdx4 knockout (KO) mouse model and a Prdx4 KO Leydig cell line to explore whether Prdx4 plays a beneficial role in spermatogenesis and spermiogenesis, and then a testicular heat stress model to investigate further the protective effect of the Prdx4 as a potential therapeutic target of male infertility. Additionally, we supplemented the mice with SLC7A11 and deferoxamine (DFO) to alleviate the testicular injury caused by Prdx4 deficiency and improve their fertility.

## 2 Materials and Methods

### 2.1 Animals

All animal experiments were approved by the Institutional Animal Care and Use Committee (IACUC‐2307053) of Nanjing Medical University Experimental Animal Department. Aged wild‐type (WT) C57BL/6 mice were purchased from Weitong Lihua Animals Co. The mice at four age stages were used as follows: (1) the 1‐week mice being at the stage of infancy (sexual infantilism), (2) the 9‐weeks mice being at the stage of young adult (sexual well‐maturation), (3) the 8 months mice being at the stage of middle age, and (4) the 12 months mice being at the stage of reproduction‐aging. Testicular heat stress was induced in those mice aged 9 weeks, using a 43°C water‐bath on bilateral testis for 15 min, one time per day in the consecutive 3 days, and 25°C water‐bath was used in the control group. Each group contains at least three independent animals. Testes were obtained from these mice 1 day after treatment.

### 2.2 Generation of Prdx4^−/y^ Male Mice

Prdx4^−/y^ male mice were generated using CRISPR/Cas9 technology with the assistance of Nanjing Biomedical Research Institute of Nanjing University, China. An sgRNA targeting Prdx4 exon 1–7 was synthesized in vitro. C57BL/6 female mice were superovulated first, then oocytes were collected from ampullae and co‐incubated with spermatozoa from C57BL/6 male mice. The pronuclear stage eggs were injected using a micromanipulator with pX330 plasmid containing Cas9 and sgRNA. The fertilized eggs were transferred into the oviducts of pseudopregnant C57BL/6J females. Next, homozygous mosaic mutant male mice were bred with C57BL/6J mice to drive homozygous mosaic mutant male mice and WT mice.

### 2.3 Mouse Genotyping

All neonatal mice were genotyped by PCR using the following primers for specific amplification of Prdx4: F: 5’‐CTGTCTCAAAATAACCACGA‐3’(sense); R:5’‐TAGCTGGATATGTTAGCACT‐3’ (antisense). PCR amplification was performed for 35 cycles (40 s at 95°C, 30 s at 59°C, 50 s at 72°C) using Recombinant Taq DNA Polymerase (R001A, TAKARA, Beijing, China). The results are shown in Figure S1.

### 2.4 Histological Examination

Testes were fixed in MDF fixative at room temperature for 48 h, followed by storage in 70% ethanol until processing for routine histology, sectioning at 4 µm thickness. For hematoxylin–eosin (HE) staining, the sections were stained with eosin and hematoxylin. Perl's Prussian blue stain was employed to assess iron precipitation in testes (G1422, Solarbio, Beijing, China).

### 2.5 Immunohistochemistry

Immunodetection of 8‐hydroxy‐2′‐ deoxyguanosine (8‐OHdG) antibody (1:200, ab26842, Abcam, Cambridge, UK), 4‐hydroxynonenal (4‐HNE) antibody (1:200, ab46545, Abcam), and endoplasmic reticulum oxidoreductase 1 (ERO1) antibody (1:200, ab177156, Abcam) were quantified using SP Rabbit&Mouse HRP Kit (DAB) (CW2069S, CW Biotec, Taizhou, China). The tissue sections were incubated with 3% hydrogen peroxide in methanol and blocked for 10 min. They were then incubated with antibody overnight in a humidified chamber at 4°C, followed by incubating with the secondary antibody with streptavidin–biotin‐horseradish peroxidase (HRP) for 10 min. After a final rinse, specific immunolabeling was examined using 3,3'‐diaminobenzidine (DAB), which was placed on the tissues for a few minutes. The sections were then washed, dehydrated, mounted, and photographed.

### 2.6 Western Blot

Total proteins were extracted from the testes using ice‐cold RIPA buffer (P0013B, Beyotime Biotechnology, Shanghai, China) supplemented with protease (P1005, Beyotime Biotechnology) and phosphatase inhibitors (P1045, Beyotime Biotechnology) according to the Bradford method. Samples were centrifuged at 12,000 *g* for 10 min at 4°C. Proteins were resolved by SDS‐PAGE, transferred to polyvinylidene difluoride (PVDF) membranes and then analyzed by immunoblot using primary antibodies including anti‐ACSL4 (1:1000, RM8098, Biodragon, Suzhou, China), anti‐ATF6 (1:2000, 24169‐1‐AP, Proteintech, Illinois, USA), anti‐β‐actin (1:1000, 66009‐1‐Ig, Proteintech), anti‐Bax (1:1000, 50599‐2‐Ig, Proteintech), anti‐Beclin (1:1000, 11306‐1‐AP, Selleck, Texas, USA), anti‐BIP (1:500, 66574‐1‐Ig, Proteintech), anti‐CAT (1:2000, SAB1405562, Sigma–Aldrich, Missouri, USA), anti‐COX2 (1:1000, RM6051, Biodragon), anti‐GAPDH (1:1000, 60004‐1‐Ig, Proteintech), anti‐GPX4 (1:1000, 52455S, CST, Massachusetts, USA), anti‐p53 (1:1000, 10442‐1‐AP, Proteintech), anti‐p62 (1:1000, E1462, Selleck), anti‐SLC7A11 (1:1000, 26864‐1‐AP, CST), anti‐SOD1 (1:1000, ab308181, Abcam), anti‐StAR (1:1000, 67130‐1‐Ig, Proteintech), anti‐Vinculin (1:1000, 13901S, CST), and anti‐XBP1S (1:1000, 24868‐1‐AP, Proteintech) antibodies. After the membranes were incubated with secondary antibodies, an ECL kit was used to detect the signals.

### 2.7 Terminal Deoxynucleotidyl Transferase‐Mediated dUTP‐Biotin Nick End Labeling (TUNEL) Assay

For the TUNEL assay, the testicular tissue slices were cleared in xylene and rehydrated. Furthermore, the slices were permeated with 0.1% TrionX‐100 at room temperature for 5 min. The apoptosis rate was detected by the TUNEL assay with the TUNEL BrightGreen Apoptosis Detection Kit (CX105, Vazyme, Nanjing, China) according to the manufacturer's instruction.

### 2.8 Measurement of Malondialdehyde (MDA)

The experimental protocol was carried out in accordance with the guidelines provided by the MDA assay kit from Beyotime (S0131). The detailed procedure was as follows: Initially, weigh 50 mg of fresh testicular tissue and add 500 µL of pre‐cooled saline to prepare the tissue homogenate. Subsequently, the homogenate was centrifuged at 12,000 *g* and the supernatant was collected. The sample was then heated at 100°C for a duration of 15 min. After cooling, a second centrifugation was performed. The supernatant was once again collected, and the absorbance was measured using a microplate reader (Multiscan FC, Thermo Fisher, Massachusetts, USA).

### 2.9 Ferrous Ion Assay

The level of testicular ferrous ions (Fe^2+^) was quantified by Ferrous Ion Content Assay Kit (AKIC004, Boxbio, Beijing, China). Briefly, process mice testicles and homogenize on ice, followed by centrifuging at 4°C 12,000 *g* for 10 min, and place the supernatant on ice. Thoroughly mix the reagents and incubate at 37°C for 10 min before adding 200 µL of chloroform and shaking vigorously for 5 min. Centrifuge at 12,000 *g* at room temperature for 10 min, then take 800 µL of the supernatant and measure the absorbance at 593 nm.

### 2.10 ELISA

To measure testosterone levels, 50µL of each standard and sample was added, followed immediately by 50µL of HRP‑conjugated antibody working solution. The mixture was incubated at 37°C for 60 min. After which, aspirate and wash the plate five times. Add 90 µL of substrate reagent and incubate for about 15 min at 37°C, followed by adding 50 µL of stop solution (JN39038, Jining Bio, Shanghai, China). Finally read the plate at 450 mm immediately.

### 2.11 Transmission Electron Microscopy (TEM)

The fresh testicular tissues were obtained and fixed at 4°C for 24 h with 2.5% glutaraldehyde. Following the standard procedure of TEM, the sample was coated with resin. The ultrathin sections were stained with lead citrate and uranyl acetate, then the images were captured by HT7700 TEM (Hitachi, Tokyo, Japan).

### 2.12 Testis Index

Mice were weighed before execution; testes and epididymis were immediately removed and weighed. The formula for calculating testis index was as follow: testis index = testicular weight (g)/mice weight (g) × 1000%.

### 2.13 Evaluation of Sperm Quality

Cauda epididymis was dissected and minced in HTF (M1135, Nanjing Aibei Biotechnology Co, Nanjing, China) at 37°C for 5 min to release and disperse spermatozoa. Sperm number, motility, and progressive rate were assessed using a computer‐assisted sperm assay (CASA) system (Video S1). Sperm drops were placed onto a slide and stained with Diff‐Quik staining solution (BA4109B, Baso Diagnostics Inc, Zhuhai, China) to assess sperm morphology according to the instructions of manufacturer.

### 2.14 Cell Culture and Treatment

MLTC‐1 is a Leydig cell line that was isolated from the testis of a male patient with a Leydig cell tumor, and serves as a versatile in vitro model for diverse research to examine how Prdx4 knockdown impact Leydig cells. MLTC‐1 cells were obtained from the Laboratory of Internal Medicine, Nanjing Medical University. MLTC‐1 cells were maintained in 1640 (A4192301, Gibco, California, USA) supplemented with 10% FBS (16000044, Thermo Fisher) and 1% penicillin‐streptomycin (C0222, Beyotime) in a 5% CO_2_ atmosphere at 37°C. To assess the effects of H_2_O_2_ (323381, Sigma–Aldrich) on MLTC‐1 cells, we selected 200 µmol H_2_O_2_ for our subsequent experiments. H_2_O_2_ was dissolved in complete medium and incubated for 8 h. The cells were stimulated by 50 mM DFO (HY‐B1625, MCE, New Jersey, USA) to inhibit ferroptosis, and transfected a plasmid with overexpression of SLC7A11 (Corues Biotechnology, Nanjing, China), according to specific experimental protocols.

### 2.15 Mitochondrial Membrane Potential (MMP) Assay

MMP was assessed using TMRE staining (C2001, Beyotime). Cells were seeded in 2 cm confocal culture dishes. After drug treatment, the TMRE working solution was added and incubated at 37°C in a cell incubator for 30 min. Following incubation, cells were washed twice with TMRE staining buffer, and then 1 mL of culture medium was added. Subsequently, cells were observed and imaged using a confocal microscope (STELLARIS, Leica, Weztlar, German), and fluorescence intensity was quantitatively analyzed.

### 2.16 Measurement of Cytoplasmic ATP Content

The measurement was performed using a luminometer (Multiscan FC, Thermo Fisher) and a commercial assay kit based on the production of fluorescence by firefly luciferase (S0027, Beyotime) following the manufacturer's recommendations. A standard curve with different concentrations from 0.1 nmol to 10 µmol was generated before the detection of ATP content in cells. For each experiment, cell lysates were loaded in 200 µL PCR tubes and then stored at 4°C until measurement of ATP content. The ATP content was finally calculated using the formula derived from the linear regression of the standard curve.

### 2.17 Cell Viability Assay

Cell viability was evaluated through the application of CCK8 (A311‐01, Vazyme). Concisely, MLTC‐1 cells were planted in a plate with 96 wells. Following a 24‐h treatment period, the viability of MLTC‐1 cells was measured using the CCK8 assay, adhering to the guidelines provided by the manufacturer. The absorbance was measured at 450 nM by using a standard instrument.

### 2.18 Determination of Total ROS

Total ROS levels in MLTC‐1 cells were determined using a ROS assay kit (S0033, Beyotime). The MLTC‐1 cells underwent treatment as directed and then diluted with 10 µmol/L DCFH‐DA using a serum‐free medium. Subsequently, MLTC‐13 cells underwent a 20‐min incubation. To eliminate the DCFH‐DA, the cells were washed thrice using PBS. The luminescence of DCF was examined through a fluorescence microscope.

### 2.19 Detection of OS Markers

OS markers, including glutathione (GSH) (A006‐2‐1, Jiancheng Biotech, Nanjing, China), 8‐OHdG (E‐EL‐0028, Elabscience, Wuhan, China), and 4‐HNE (E‐EL‐0128, Elabscience) were analyzed using reagent kits. Each procedure followed the manufacturer's recommendations. The absorbance levels were calculated independently.

### 2.20 MitoTracker

Assessment of mitophagy in cells’ mitochondria were labeled with a 0.1 µM MitoTracker Red CMXRos probe (9082S, CST) emitting in the red (561 nm excitation, 590 nm emission) channel.

### 2.21 Caspase‐3 Activity Analysis

To examine the activity of caspase‐3 in Leydig cells, a caspase‐3 activity assay kit (AKPR027, Boxbio) was used according to the manufacturer's instructions.

### 2.22 Evaluated the Level of Lipid ROS

C11‐Bodipy 581/591 probe (Beyotime, S0043) was employed to measure the level of lipid ROS in testicular Leydig cells, following the instructions provided in the manual.

### 2.23 Measurement of Intracellular Iron Levels by FerroOrange

Intracellular Fe^2+^ was assessed using FerroOrange (36104S, CST) according to the manufacturer's instruction. Briefly, cells seeded in confocal dishes were stained with HBSS buffer containing 1 µM FerroOrange reagent for 30 min at 37°C incubator with 5% CO_2_. Fluorescence images were captured by the Laser Scanning Confocal Microscope STELLARIS (Leica).

### 2.24 Statistical Analysis

The specific sample sizes (*n* numbers) were provided in figure legends. Each experiment was conducted independently at least three times. SPSS 20.0 statistical software was used to analyze the data. The count data were expressed as mean ± standard deviation, and the independent sample *t* test was used for analysis. One‐way ANOVA was followed by Tukey's post hoc test among three or more groups. The exact *p* values were detailed in the corresponding figure legends. *p* < 0.05 indicates that the difference is statistically significant.

## 3 Results

### 3.1 The Decreased Fertility in Prdx4^−/y^ Male Mice

**FIGURE 1:**
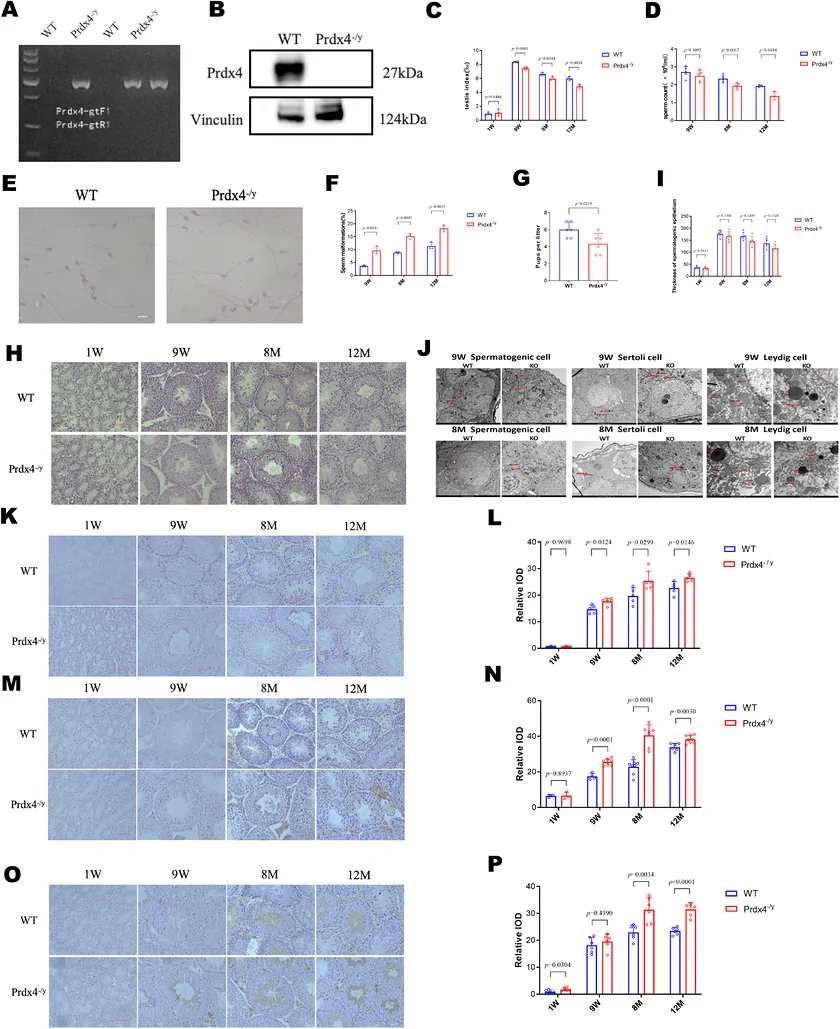
Establishment of the Prdx4^−/y^ mouse model and acceleration of testicular aging in Prdx4^−/y^ mice. (A) PCR genotyping of mice. Agarose gel analysis of PCR products amplified from DNA isolated from tails of male mice. These primers generated a 700‐bp product. Genes from wild‐type mice (Lanes 1 and 3) and Prdx4^−/y^ mice (Lanes 2 and 4) were amplified with specific primers. (B) Immunoblotting analysis of Prdx4 protein expression in the wild‐type and Prdx4^−/y^ mice. The blot was probed with an anti‐Prdx4 antibody. Vinculin was used as a loading control. (C) The testis index of the wild‐type and Prdx4^−/y^ mice aged at 1 week, 9 weeks, 8 months, and 12 months (*n* = 4). (D) Comparison of sperm count in epididymal fluid from wild‐type and Prdx4^−^ *^/^* ^y^ mice at the indicated ages (*n* = 3). (E) The Diff‐Quik staining of spermatozoa collected from cauda epididymis of wild‐type and Prdx4^−^ *^/^* ^y^ mice at the indicated ages. Scale bar = 10 µm. (F) Malformed spermatozoa were counted and the proportion between wild‐type and Prdx4^−^ *^/^* ^y^ mice were compared (*n* = 3). (G) Note that 9‐week‐old wild‐type and Prdx4^−^ *^/^* ^y^ mice were caged with WT females for 3 months; litter size showed a downward trend in Prdx4^−^ *^/^* ^y^ mice (*n* = 6). (H, I) The H&E staining of testes and from wild‐type and Prdx4^−/y^ mice (*n* = 5). Scale bar = 200 µm. (J) Representative images of transmission electron microscopy (TEM) images of spermatogenic cells, Sertoli cells, and Leydig cells in 9‐week‐old and 8‐month‐old wild‐type and Prdx4^−/y^ mice. (K–N) The cellular localization of oxidative damage‐related factors, (K, L) 4‐HNE and (M, N) 8‐OHdG were observed using immunohistochemistry. The Prdx4^−/y^ group showed significantly increased expression of 8‐OHdG and 4‐HNE in middle‐aged and aging mice (*n* = 3). Scale bar = 200 µm. (O, P) The cellular localization of ERO1 (*n* = 3). Scale bar = 200 µm. All data were shown as means ± SEMs from at least three independent animals. Statistical analysis was performed using one‐way ANOVA with Tukey post‐hoc test. ns indicates non‐significant. *p < 0.05; **p < 0.01; ***p < 0.001.

CRISPR/Cas9 system was used to develop the male Prdx4^−/y^ C57BL/6J mice by deleting Prdx4 exon 1–7 (Figure S1A). Genotypes were confirmed by PCR (Figure 1A) (Figure S1B) and western blot (Figure 1B, seen in Supporting Information). Besides, a brief off‐target assessment was shown in Table S1 to identify potential off‐target sites and evaluate the specificity of the CRISPR‐Cas9 gene editing system. It was shown that Prdx4 gene was absent absolutely.

To explore the impact of Prdx4 KO on the male fertility, we compared the testis index (the index of testicle weight/body weight), sperm count, and sperm malformation rate between the Prdx4^−/y^ mice and the WT mice in the respective four age subgroups, including (1) the 1‐week infancy mice, (2) the 9‐weeks adult (young) mice, (3) the 8‐months middle‐aged mice, and (4) the 12‐months aging mice. Comparing the Prdx4^−/y^ infancy mice with the WT infancy mice, there were no differences in above parameters. Interestingly, there were significant differences in the testis index (Figure 1C) and sperm count (Figure 1D) between the Prdx4^−/y^ adult mice and the WT adult mice, which were approximately 20% lower in the Prdx4^−/y^ adult mice (both *p* < 0.05). What should be paid attention to is that the differences in the testis index and sperm count were very significant between the Prdx4^−/y^ middle‐aged mice and the WT middle‐aged mice, or between the Prdx4^−/y^ aging mice and the WT aging mice, with the decreased degree of over 35% respectively (all *p* < 0.05) (Figure 1C,D). Correspondingly, the sperm malformation rates in the Prdx4^−/y^ mice at the middle and aging stages were significantly increased when compared with their controls (both *p* < 0.05) (Figure 1E,F) (Video S1). As for fertility in Prdx4^−^ *^/^* ^y^ mice, the young adult male WT mice and Prdx4^−^ *^/^* ^y^ mice were caged with the WT female mice aged 3 months, and the litter size showed a downward trend in the Prdx4^−^ *^/^* ^y^ mice (Figure 1G).

The histological examination of testes showed that the seminiferous epithelium of Prdx4^−/y^ mice was almost the same as the control (Figure 1H). Thickness of spermatogenic epithelium was significantly decreased in the Prdx4^−/y^ mice aged 8 months and 12 months (*p* < 0.05) (Figure 1I). TEM showed that in 9‐week and 8‐month of Prdx4^−/y^ male mice, the mitochondrial matrix became diluted with a reduced electron density, and even appeared vacuolated. Furthermore, the nuclei of spermatogenic cells carried scanty electron‐dense bodies, while the residual bodies were frequently observed in the cytoplasm of Sertoli cells (Figure 1J).

### 3.2 OS and ERS in the Prdx4^−/y^ Mice

To evaluate the antioxidant effect of Prdx4 in testes, the expression levels of 4‐HNE (a lipid hydroperoxide) (Figure 1K) and 8‐OHdG (a DNA oxidation biomarker) (Figure 1M) were measured in the Prdx4^−/y^ and WT mice. In testicular somatic cells, 4‐HNE exhibited strong staining, whereas weak staining was observed in spermatogenic cells. In the peripheral somatic cells, 4‐HNE was expressed strongly, and in the spermatogenic cells, it was expressed weakly. The expression level of 4‐HNE was significantly increased in all Prdx4^−/y^ adult mice (*p* < 0.01). Positive staining of 8‐OHdG was found mainly in spermatogenic cell, spermatids, and peripheral somatic cells, especially in spermatids. The middle‐aged and aging Prdx4^−/y^ mice displayed significantly higher expression of 8‐OHdG, compared with the age‐matched WT mice (*p* < 0.01). The changes of 4‐HNE and 8‐OHdG indicated that the Prdx4^−/y^ mice at the middle‐aged and aging stages showed a higher level of OS (Figures 1L,N).

ERO1 as a main protein forming disulfide bond in ER may partially compensate Prdx4's function. The expressions of ERO1 in the testicular tissues of Prdx4^−/y^ mice and WT mice were compared by IHC. The ERO1 expression was increased with age (*p* < 0.01) (Figures 1O,P). The significant difference in the ERO1 expression was observed in the Prdx4^−/y^ middle‐aged and aging mice compared with the WT mice, which were approximately 20% higher in the Prdx4^−/y^ mice.

### 3.3 Poorer Fertility in the Adult Prdx4^−/y^ Mice After Heat Stress

**FIGURE 2:**
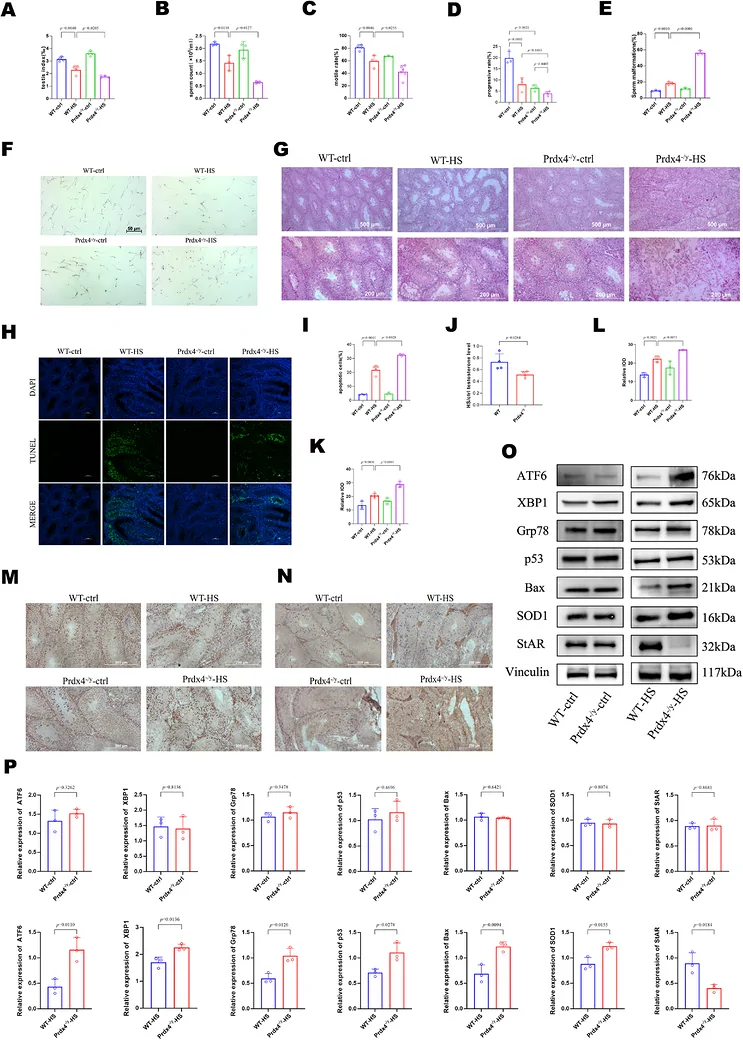
Heat stress reproductive damage and heat stress‐induced OS in Prdx4^−/y^ male mice. (A) The testis index comparing the control group and heat‐stress group in wild‐type and Prdx4^−/y^ adult mice (*n* = 4). (B–D) Cauda epididymis was dissected and minced in HTF at 37°C for 5 min to release and disperse spermatozoa. Then, CASA analysis of spermatozoa from cauda epididymis of wild‐type and Prdx4^−/y^ adult mice were performed. Sperm count, motile rate, and progressive rate were documented (*n* = 3). (E, F) The Diff‐Quik staining of spermatozoa from cauda epididymis, and the proportion of malformed spermatozoa were calculated in each group (*n* = 3). Scale bar = 50 µm. (G) The H&E staining of testes from control and heat‐stress group in wild‐type and Prdx4^−/y^ adult mice. Scale bars = 500 µm and 200 µm. (H, I) TUNEL staining and the percentage of positive cells in the testicular sections from heat‐stress and control group in wild‐type and Prdx4^−/y^ adult mice (*n* = 3). Scale bar = 50 µm. (J) Detection of total testosterone using ELISA in mice serum (*n* = 4). (K–N) Increased oxidative damage was found in adult Prdx4^−/y^ mice under heat‐stress, which showed significantly increased expression of (K, M) 4‐HNE, as well as (L, N) 8‐OHdG. Protein expression was quantitatively analyzed (*n* = 3). Scale bar = 200 µm. (O, P) The expression of antioxidant enzymes and ERS pathway‐associated factors XBP1, ATF6, Grp78, p53, and Bax in heat‐stress induced and control group (*n* = 3). Results were presented as mean ± SEMs from three independent animals. Statistical analysis was performed using one‐way ANOVA with Tukey post‐hoc test. ns indicates non‐significant. *p < 0.5; **p < 0.01; ***p < 0.001; ****p < 0.00001.

It was true that compared with WT adult mice, the Prdx4^−/y^ adult mice didn't show a significant decrease in fertility, by checking testis index, sperm count, and sperm malformation rate. To explore whether there is potential imbalance between oxidation and antioxidation in the adult Prdx4^−/y^ mice, the testes 43°C water‐bath was used in the 9‐week‐old mice to induce a heat stress. The testis index and sperm count were significantly decreased, and the sperm malformation rate increased in both Prdx4^−/y^ adult mice and WT mice (all *p* < 0.05). After heat stress, the Prdx4^−/y^ mice had a greater decline degree in the testis index than the WT mice (Figure 2A). Importantly, Prdx4^−/y^ mice showed a substantial decrease in sperm count (Figure 2B) and motile rate than the WT mice (Figure 2C), as well as the progressive rate (PR) (all *p* < 0.05) (Figure 2D) (Video S1); meanwhile, heat stress reinforces these trends. As vividly shown in Figure 2E,F, the sperm malformations were observed in the Prdx4^−/y^ mice and WT mice under heat stress, which showed the ample sign of asthenoteratozoospermia including absent, short, and coiled flagella, angulation, and irregular caliber. In testicular histopathology, the WT mice after the heat stress treatment were represented vacuolization and multinucleated giant cells (MGC) appearance in the seminiferous tubule, the disorder of cell arrangement. In the Prdx4^−/y^ mice, these changes were much more serious. The cell arrangement of seminiferous tubules worsened to the extent that it was completely disordered, and spermatid almost disappeared (Figure 2G).

### 3.4 Response to Heat Stress in the Adult Prdx4^−/y^ Mice

The apoptosis index (rate of TUNEL‐positive cells) of testicular tissues were significantly increased in both Prdx4^−/y^ adult mice and WT mice after the treatment of testicular heat stress (both *p* < 0.01) (Figure 2H,I). The increased degree of apoptosis index in the Prdx4^−/y^ adult mice was significantly higher than that in the WT adult mice. The expression of Bax protein was more significantly increased in the Prdx4^−/y^ adult mice after heat stress, indicating that Prdx4 may partially protect the spermatogenic cells against the apoptosis induced by heat stress (Figure 2O).

ELISA test showed that the level of serum testosterone was decreased in the WT mice after heat stress, and that this decrease was more significant in the Prdx4^−/y^ mice after heat stress (*p* < 0.05) (Figure 2J). The expression of steroidogenic acute regulatory protein (StAR) was significantly decreased, which was parallel with the testosterone level, further suggesting that Prdx4 may play a protective role in maintaining function of testicular Leydig cells (Figure 2O, seen in Supporting Information).

After the treatment of heat stress, the expression of 4‐HNE (Figure 2K,M) and 8‐OHdG (Figure 2L,N) was significantly increased (both *p* < 0.01). Compared with the WT mice, the Prdx4^−/y^ mice showed more significant changes in the expressions of 8‐OHdG and 4‐HNE, suggesting anabatic disorder of OS. To evaluate the response of ERS, the expressions of ERS‐related apoptotic markers in testicular tissues were measured by western blotting (Figure 2O,P). The expressions of XBP1, ATF6, Grp78, and p53 were significantly increased in both Prdx4^−/y^ mice and WT mice after heat stress, and the change degree in Prdx4^−/y^ mice was higher than that was in WT group (*p* < 0.05).

### 3.5 Effects of Prdx4 on the Biochemical Events of Ferroptosis

**FIGURE 3:**
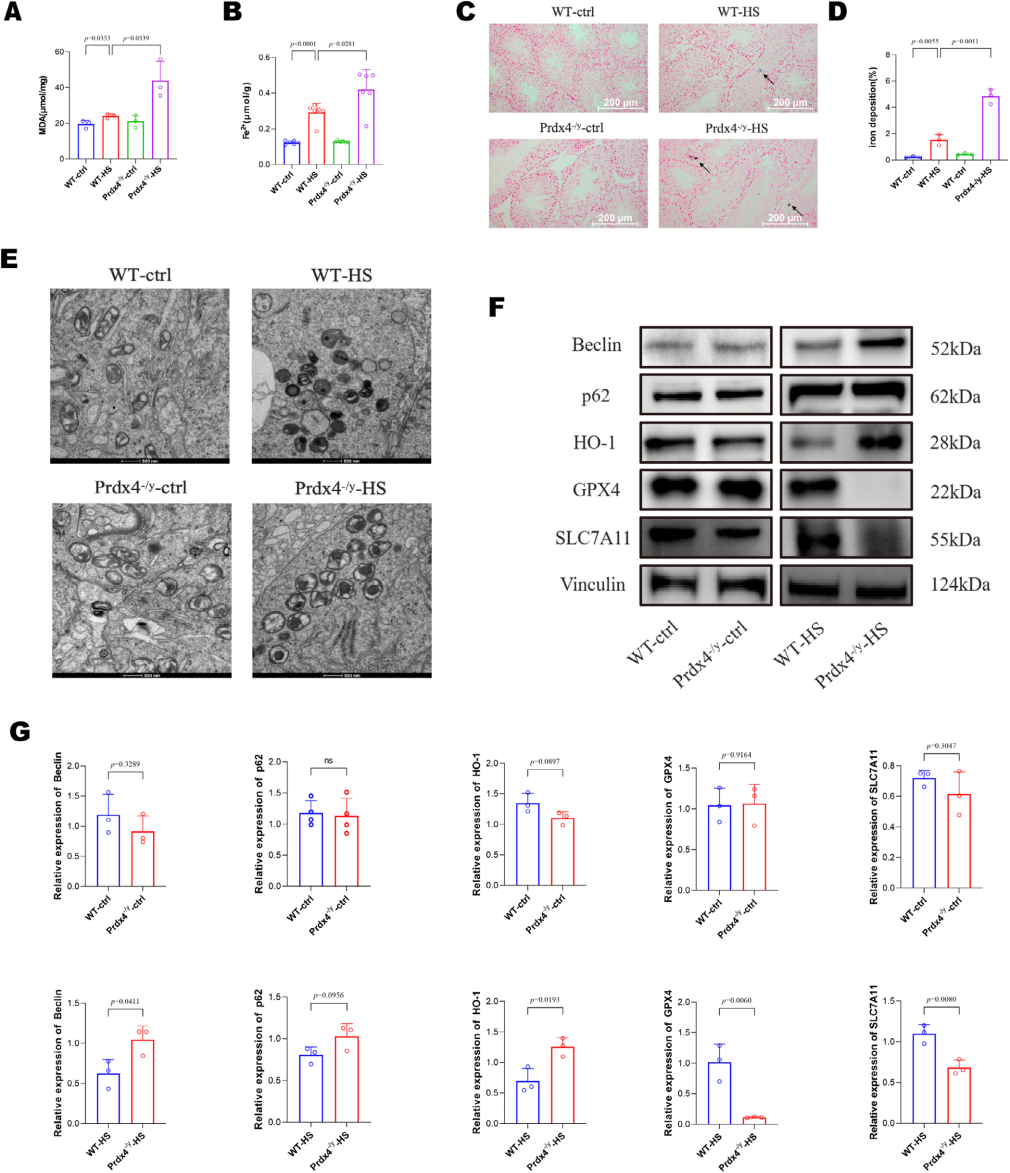
Heat stress‐induced ferroptosis and mitochondrial autophagy in the Prdx4^−/y^ adult mice. (A) Malondialdehyde (MDA) content in testes (*n* = 3). (B) Analysis of intracellular Fe^2+^ in testes (*n* = 6). (C, D) Representative figures of Perl's Prussian blue staining (*n* = 3) and quantification of iron deposition. Scale bar = 200 µm. (E) Representative images of TEM images in heat‐stress and control group in wild‐type and Prdx4^−/y^ adult mice (*n* = 3). Scale bar = 500 nm. (F, G) The expression of ferroptosis, including GPX4 and SLC7A11 in testes. Protein expression was quantitatively analyzed (*n* = 3). All data are shown as means ± SEMs from three independent animals. Statistical analysis was performed using one‐way ANOVA with Tukey post‐hoc test. ns indicates non‐significant. *p < 0.05; **p < 0.01; ***p < 0.001.

Ferroptosis of testicular cells in Prdx4^−/y^ adult mice were evaluated by iron accumulation and lipid peroxidation. Quantitative analysis of MDA (an endogenous genotoxic product of lipid peroxidation) revealed that the KO of Prdx4 significantly facilitated the heat‐stress induced lipid peroxidation in mice testes (Figure 3A). According to intracellular Fe^2+^ and Perl's Prussian blue staining, the testes of the Prdx4^−/y^ adult mice under heat stress accumulated more iron than the WT group (Figures 3B–3D). Furthermore, the TEM images revealed that the Prdx4^−/y^ adult mice under heat‐stress exhibited shrunken mitochondria with increased membrane density when compared with the WT group, which was accompanied by cristae fragmentation (Figure 3E). According to the immunoblotting results, Prdx4^−/y^ adult mice under heat stress noticeably downregulated the expressions of glutathione peroxidase 4 (GPX4) and solute carrier family 7 member 11 (SLC7A11) in testes (Figure 3F,G, seen in Supporting Information). Altogether, these findings demonstrated that Prdx4 should be a positive regulator of ferroptosis.

### 3.6 Ablation of Prdx4 Aggravated Cytotoxicity and Mitochondria‐Derived OS in H_2_O_2_‐Induced MLTC‐1 Cells

CRISPR/Cas9 system was used to develop the male Prdx4^−/y^ Leydig cells by deleting Prdx4 exon 1–7. Genotypes were confirmed by western blot (Figure S2A,B). It was shown that Prdx4 gene was downregulated. To further investigate how KO of Prdx4 disrupts spermatogenesis in Leydig cells, MLTC‐1 cells were cultured in medium containing 200 µmol H_2_O_2_ for 8 h for our subsequent experiments. ELISA test showed that the level of testosterone was decreased in the MLTC‐1 cells after H_2_O_2_ treatment, and that this decrease was more significant in the Prdx4^−/y^ Leydig cells (Figure S2C). CCK8 assay was used in this study. As shown in Figure S2D, H_2_O_2_ significantly decreased cell survival rate of Leydig cells to 53.2%, while KO of Prdx4 significantly facilitated the cytotoxicity under the treatment of H_2_O_2_. We found that knockdown of Prdx4 increased the level of caspase‐3 compared with the H_2_O_2_ group (Figure S2E). Moreover, the apoptosis index was significantly increased in both WT and Prdx4^−/y^ Leydig cells after the treatment of hydrogen peroxide (both *p* < 0.01) (Figure S2F,G). The data suggested that the Prdx4 knockdown remarkably induced cell apoptosis in the H_2_O_2_‐induced Leydig cells.

**FIGURE 4:**
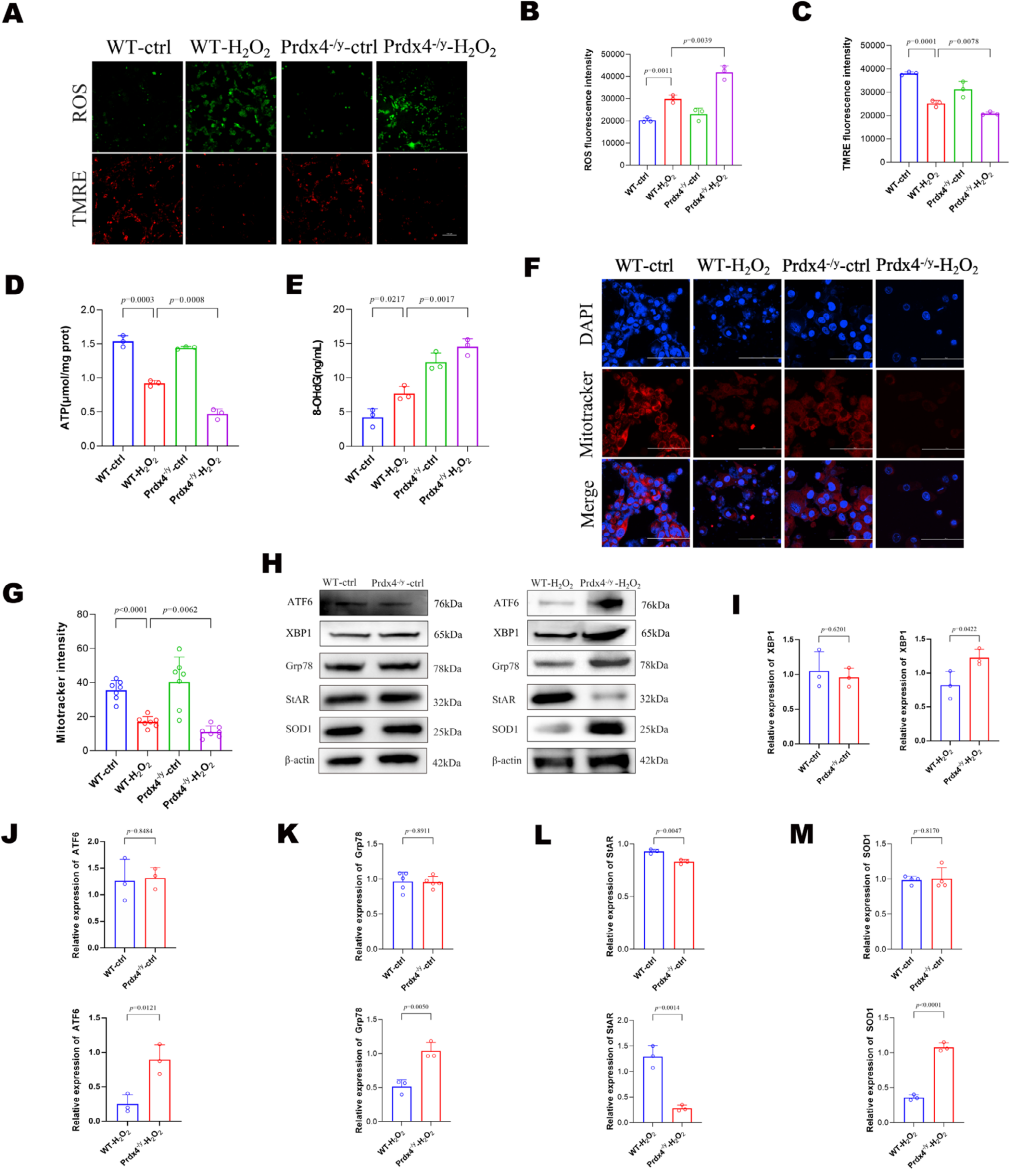
Ablation of Prdx4 aggravated mitochondria‐derived oxidative stress in H_2_O_2_‐induced MLTC‐1 cells. Prdx4^−/y^ and wild‐type MLTC‐1 were cultured with H_2_O_2_ (200 µmol) for 8 h for our subsequent experiments. Cells without any treatment were used as blank controls. (A, B, C) ROS levels and MMP levels in H_2_O_2_‐induced MLTC‐1 cells (*n* = 3). Scale bar = 100 µm. (D) Levels of ATP in MLTC‐1 cells followed by H_2_O_2_ treatment (*n* = 3). (E) Level of 8‐OHdG in MLTC‐1 cells (*n* = 3). (F) Representative images of mitochondrial distribution in the different groups. Scale bar = 100 µm. (G) Fluorescence intensity of MitoTracker Red staining was shown. (H–M) The expression of antioxidant enzymes and ERS pathway‐associated factors including XBP1, ATF6, Grp78, SOD1, and StAR in H_2_O_2_‐treated and control group (*n* = 3). All data are shown as means ± SEMs from at least three independent experiments. Statistical analysis was performed using one‐way ANOVA with Tukey post‐hoc test. ns indicates non‐significant. * *p* < 0.05; **p < 0.01; ***p < 0.001.

As shown in Figure 4A–C, the results indicated that H_2_O_2_ significantly increased the level of ROS and decreased MMP levels. After KO of Prdx4, ROS levels were then significantly increased, while MMP levels demonstrated a significant decline. We found that H_2_O_2_ treatment caused lower ATP contents (Figure 4D), suggesting that the Prdx4 KO downregulated ATP production in MLTC‐1 cells. Furthermore, we detected the mitochondrial quantity using MitoTracker Red. As shown in Figure 4F,G, H_2_O_2_‐exposed cells showed decreased mitochondrial mass, as seen in the decline of the MitoTracker Red fluorescence signals. Consistent with our findings in mice testis, knockdown of Prdx4 significantly triggered mitochondria‐dependent cell apoptosis by decreasing MMP and reducing mitochondria‐derived ROS production. Following treatment with 200 µmol H_2_O_2_, SOD1 showed notable reductions as antioxidant indicators (Figure 4H,M, seen in Supporting Information). It was also observed that there was a notable increase in 8‐OHdG level (Figure 4E). According to immunoblotting results, the Prdx4^−/y^ Leydig cells under OS noticeably downregulated the expressions of StAR, while the expressions of ERS markers XBP1, ATF6, and Grp78 were increased (Figure 4H–L). The results verified that the ablation of Prdx4 induced triggered cytotoxicity and OS in MLTC‐1 cells.

### 3.7 Prdx4 KO Aggravated H_2_O_2_‐Induced Ferroptosis

**FIGURE 5:**
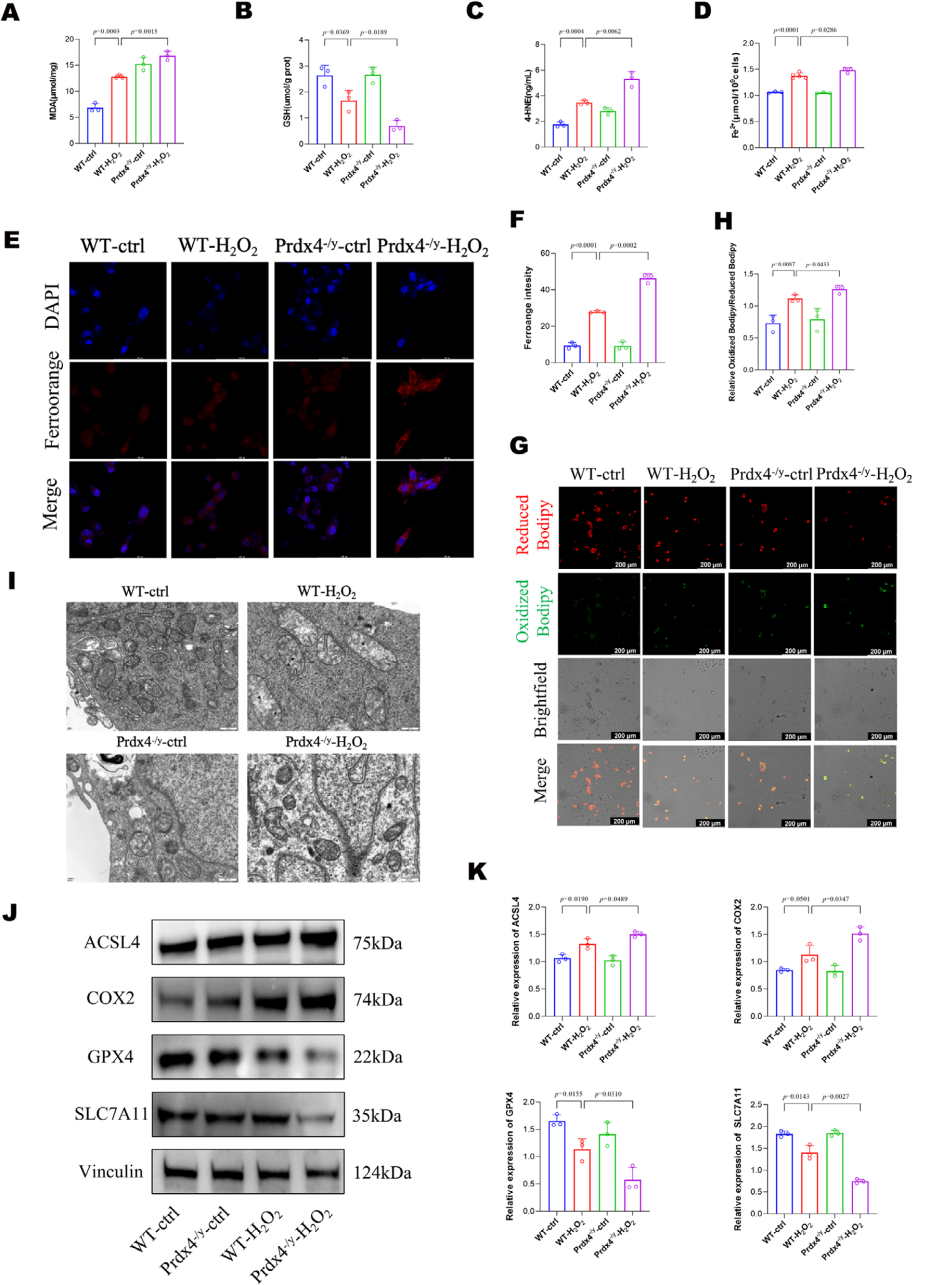
Prdx4 knockout aggravated H_2_O_2_‐induced ferroptosis. (A, B) Malondialdehyde (MDA) content and GSH levels in H_2_O_2_‐induced MLTC‐1 cells (*n* = 3). (C) Increased oxidative damage was found in H_2_O_2_‐induced MLTC‐1 cells, which showed significantly increased expression of 4‐HNE (*n* = 3). (D) Analysis of intracellular Fe^2+^ in MLTC‐1 cells (*n* = 3). (E, F) Confocal microscope images and fluorescence intensity of FerroOrange‐stained MLTC‐1 cells treated with H_2_O_2_ (*n* = 3). Scale bar = 100 µm. (G, H) Levels of lipid ROS in Prdx4^−/y^ MLTC‐1 cells treated with H_2_O_2_ (*n* = 3). Scale bar = 200 µm. (I) Representative images of TEM images in heat‐stress and control group in wild‐type and Prdx4^−/y^ MLTC‐1 cells (*n* = 3). Scale bar = 500 nm. (J, K) The expression of ferroptosis factors ACSL4, COX2, GPX4, and SLC7A11 in wild‐type and Prdx4^−/y^ MLTC‐1 cells (*n* = 3). Protein expression was quantitatively analyzed. All data are shown as means ± SEMs from at least three independent experiments. Statistical analysis was performed using one‐way ANOVA with Tukey post‐hoc test. ns indicates non‐significant. *p < 0.05; **p < 0.01; ***p < 0.001.

We next examined the effects of Prdx4 on the biochemical events of ferroptosis, including iron accumulation and lipid peroxidation. Quantitative analysis of MDA revealed that the KO of Prdx4 significantly accelerated the H_2_O_2_‐induced lipid peroxidation in Leydig cells (Figure 5A). GSH, as a ferroptosis indicator, showed notable reduction when exposed to H_2_O_2_ in the Prdx4^−/y^ MLTC‐1 cells (Figure 5B). The expression levels of 4‐HNE were measured in the Prdx4^−/y^ and WT Leydig cells. A significant increase of 4‐HNE expression was also found in the cells after H_2_O_2_ treatment (Figure 5C) (*p* < 0.01). According to intracellular Fe^2+^, Prdx4^−/y^ Leydig cells under OS accumulated more iron than the WT group (Figure 5D). A larger amount of Fe^2+^ was shown in Leydig cells stained with FerroOrange than that of control cells after H_2_O_2_ treatment, and the KO of Prdx4 exacerbated this trend (Figure 5E,F). Moreover, lipid ROS levels measured by C11‐Bodipy increased significantly in the Prdx4^−/y^‐H_2_O_2_ group (Figure 5G,H). Furthermore, the TEM images revealed that the Prdx4^−/y^ Leydig cells under OS, when compared with the WT group, exhibited decreased volume in most of the mitochondria in the cytoplasm; higher electron density of the mitochondrial membrane; reduced, thickened, or blurred inner ridges of the mitochondria; and mild dilation of the ER (Figure 5I). According to immunoblotting results, the Prdx4^−/y^ Leydig cells under OS noticeably downregulated the expressions of GPX4 and SLC7A11, while the expressions of Cyclooxygenase‐2 (COX2) and Acyl‐CoA synthetase long chain family member 4 (ACSL4) were both increased (Figure 5J,K, seen in Supporting Information). Altogether, these findings demonstrated that Prdx4 was a positive regulator of ferroptosis.

### 3.8 DFO Prevented Iron Overload‐Induced Testicle Injury in Prdx4 KO Leydig Cells

**FIGURE 6:**
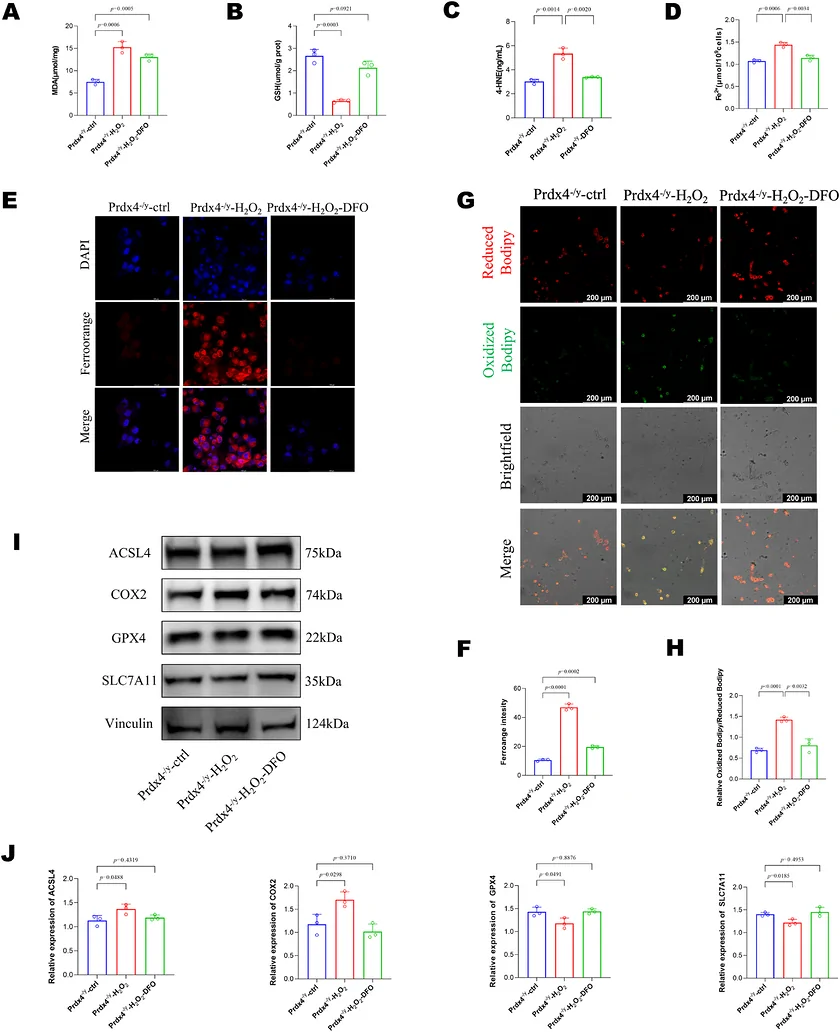
Deferoxamine (DFO) rescues iron overload‐induced testicle injury in Prdx4 knockout Leydig cells, as well as overexpressing SLC7A11. (A–D) MDA content, GSH levels, and 4‐HNE and Fe^2+^ levels were measured in Prdx4^−/y^ MLTC‐1 cells, H_2_O_2_‐induced Prdx4^−/y^ MLTC‐1 cells, and H_2_O_2_‐induced Prdx4^−/y^ MLTC‐1; DFO‐treated cells (*n* = 3). (E, F) Confocal microscope images of FerroOrange‐stained in H_2_O_2_‐induced Prdx4^−/y^ MLTC‐1 cells treated with DFO (*n* = 3). Scale bar = 100 µm. (G, H) Level of oxidized/reduced Bodipy in Prdx4^−/y^ MLTC‐1 cells treated with DFO (*n* = 3). Scale bar = 200 µm. (I, J) The expression of ferroptosis factors ACSL4, COX2, GPX4, and SLC7A11 in DFO‐treated Prdx4^−/y^ MLTC‐1 cells (*n* = 3). Protein expression was quantitatively analyzed. Statistical analysis was performed using one‐way ANOVA with Tukey post‐hoc test. ns indicates non‐significant. *p < 0.05; **p < 0.01; ***p < 0.001.

Next, to test our hypothesis that ferroptosis plays a pathogenic role in OS in Prdx4^−/y^ Leydig cells, we tested whether DFO, a selective ferroptosis inhibitor, could protect against testicle injury by treating Prdx4^−/y^ Leydig cells. In the Prdx4^−/y^ Leydig cells, DFO treatment significantly protected H_2_O_2_‐treated Leydig cells from MDA, GSH, 4‐HNE levels (Figure 6A–C), and Fe^2+^, as shown in the Fe^2+^ level and FerroOrange intensity (Figure 6D–F). Subsequently, DFO addition significantly mitigated lipid ROS levels in the H_2_O_2_‐treated group (Figure 6G–H). In addition, the DFO‐treated Prdx4^−/y^ Leydig cells had significantly reduced the expressions of the genes associated with ferroptosis under OS (i.e., reduced GPX4 and SLC7A11, elevated COX2 and ACSL4 protein expression) compared to Prdx4^−/y^ Leydig cells (Figure 6I, J, seen in Supporting Information).

### 3.9 Testicle Ferroptosis in H_2_O_2_‐Treated Prdx4^‐/y^ Leydig Cells Was Rescued by Overexpressing SLC7A11

**FIGURE 7:**
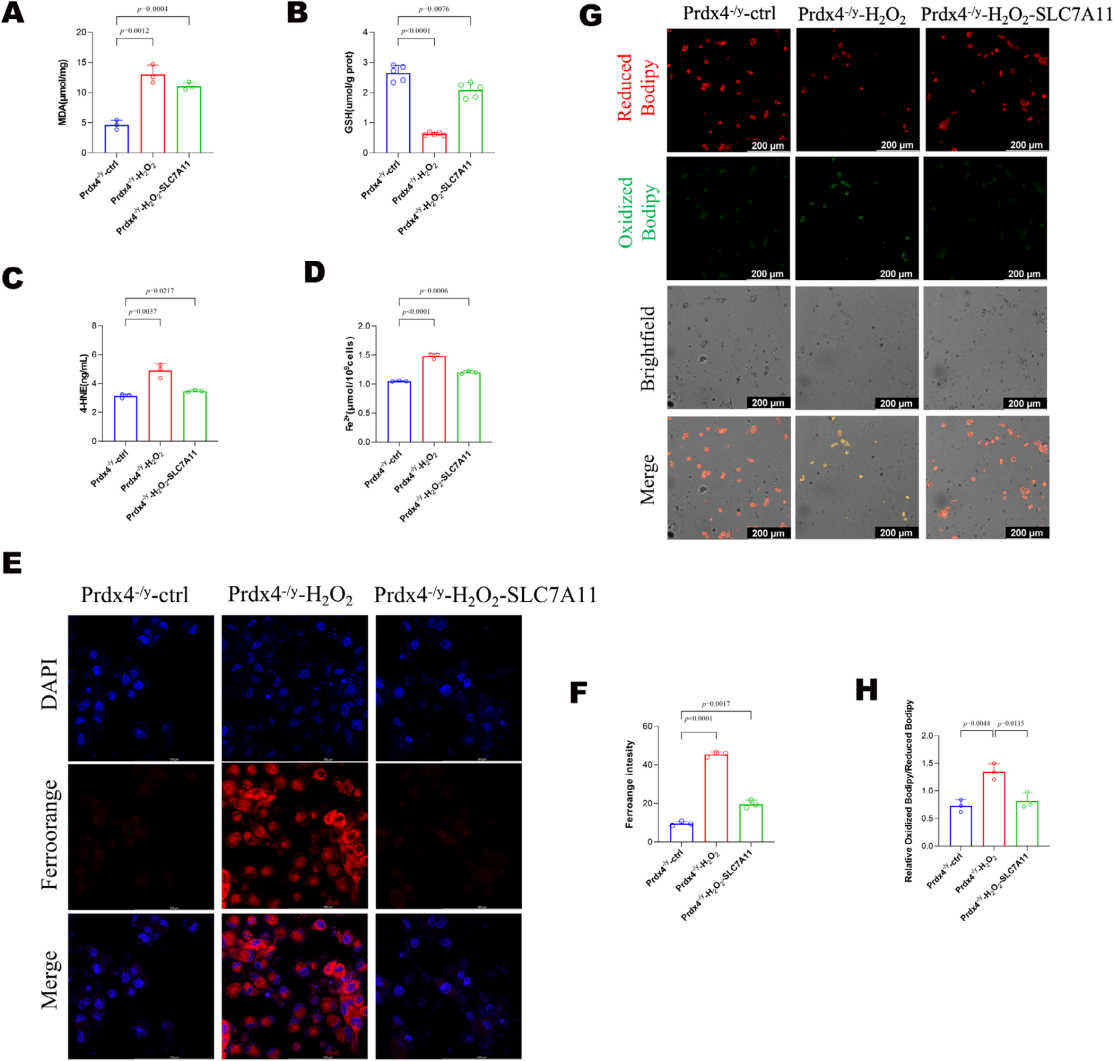
Overexpressing SLC7A11 rescues iron overload‐induced testicle injury in Prdx4 knockout Leydig cells. (A–D) Summary of the MDA, GSH, 4‐HNE, and Fe^2+^ levels in Prdx4^−/y^ MLTC‐1 cells, H_2_O_2_‐induced Prdx4^−/y^ MLTC‐1 cells, and H_2_O_2_‐induced Prdx4^−/y^ MLTC‐1; SLC7A11‐overexpressing cells (*n* = 3). (E, F) Confocal microscope images of FerroOrange‐stained in H_2_O_2_‐induced Prdx4^−/y^ MLTC‐1 cells overexpressing SLC7A11 (*n* = 3). Scale bar = 100 µm. (G, H) Level of oxidized/reduced Bodipy in Prdx4^−/y^ MLTC‐1 cells overexpressing SLC7A11 (*n* = 3). Scale bar = 200 µm. All data are shown as means ± SEMs from at least three independent experiments. Statistical analysis was performed using one‐way ANOVA with Tukey post‐hoc test. ns indicates non‐significant. *p < 0.05; **p < 0.01; ***p < 0.001.

Previous studies showed that SLC7A11 plays an important role in regulating ferroptosis in certain cancer cells. We therefore tested whether the overexpression of SLC7A11 reduced ferroptosis of Leydig cells. As expected, the overexpressing SLC7A11 in Prdx4^−/y^ Leydig cells restored the levels of MDA and GSH (Figure 7A,B), reduced ferroptosis (i.e., decreased FerroOrange intensity and 4‐HNE levels) under OS (Figure 7C–F) and the levels of lipid ROS levels (Figure 7G–H). Taken together, these findings were consistent with our results obtained by pharmacologically blocking ferroptosis, suggesting that the overexpressing SLC7A11 in testicle Leydig cells suppressed ferroptosis, and protected against testicle aging, thereby improving the testicle function in the Prdx4‐deficient Leydig cells under iron overload conditions.

## 4 Discussion

Prdx4 serves as an endogenous antioxidant in mitigating intracellular OS [25], and a vital protector against ERS [26]. In the present study, we explored the beneficial roles of Prdx4 in sperm production by alleviating the OS and ERS and blocking ferroptosis. We found that KO of Prdx4 affected the spermatogenesis and Leydig's function following the process of testicular aging and heating stress, by the related OS, ERS, and ferroptosis. Our results suggested that Prdx4 may function as a protective factor of testes against oxidative damage.

There exist two localizations of Prdx4, one of which is located in ER [24]. Under the condition of ERS, the finely‐tuned homeostasis of calcium ion within ER was perturbed, and excess calcium ion was secreted from ER to cytoplasm [27]. This activation disrupted the normal function of the mitochondrial respiratory chain due to the overburdened calcium within mitochondria, thereby promoting the generation of ROS [28]. Prdx4 is also a critical factor of the oxidative folding of functional proteins in ER. The Prdx4 located in ER just plays these key roles in effectively maintaining the ER homeostasis by removing H_2_O_2_. Additionally, the Prdx4 located in cytoplasm, even secreted to the extracellular matrix, can reduce H_2_O_2_ and mitigate ROS level through stress kinases [25]. Our previous study found that the Prdx4 secreted by cumulus cells was a new favorable regulator that improved the quality of in vitro maturation (IVM) oocytes and enhanced the developmental competence of IVM oocytes by preventing the apoptosis caused by OS [29]. As an intracellular antioxidant enzyme, Prdx4 is abundantly expressed in testicular cells, including spermatogonia, spermatocyte, Sertoli cells, and Leydig cells [20]. Prdx4t, a testis‐specific isoform of the Prdx4 family, began to express after 4 weeks of age and mainly in sperm cells [30], suggesting the importance of Prdx4 in spermatogenesis [19].

In the present study, we explored the protective effect of Prdx4 in testes. We found that the Prdx4 KO adult mice had fewer offspring per litter, although without significant differences in sperm counts, sperm morphology, OS, or ERS between the Prdx4^−/y^ and WT adult mice before aging. A previous study reported that the Prdx4 KO mice exhibited the increased susceptibility to OS, leading to the reduced sperm counts and the impaired fertility [31]. Other studies found that during the process of testicular aging, the level of OS rise with age [32]. Notably, OS plays a pivotal role in the physiological cellular aging and the accumulated ROS, coupled with the imbalance in antioxidant defenses, which constitutes a key pathophysiological mechanism underlying testicular aging [33]. In this study, considering MLTC‐1 is a tumor‐derived cell line, all key enzymes and pathways in steroid synthesis exhibited the same as mouse primary Leydig cells. The core difference lies in that MLTC‐1 has a stronger tolerance to OS while the antioxidant homeostasis and hormone secretion of primary cells all conform to physiological characteristics.

OS significantly impacts the testicular microenvironment, which impairs spermatogenesis and results in the reduced sperm quantity and quality [34]. Excessive ROS leads to the DNA chain damage in spermatozoa [35], considering that sperm membrane is rich in the PUFAs and highly sensitive to OS [36]. Furthermore, excessive ROS damages sperm capacitation and acrosome reaction, leading to oligospermia and asthenospermia in men [37]. Meanwhile, OS may break the Leydig cells located in interstitial tissue, and may be damaged, adversely impacting testosterone production [38, 39]. Our team has previously explored the methods for establishing testicular heat stress models and the effects based on previous literature [40, 41]. In the present study, we found that the spermatogenesis in the Prdx4^−/y^ adult (young) mice is almost normal, and that the Prdx4^−/y^ adult mice after heat stress treatment showed significant reductions in the testis index, sperm progressive rate, and sperm motile rate while there was elevation in the apoptosis rate and sperm malformation rate. Apart from sperm quality, however, in future work, more data including copulatory plug rates, pregnancy rate, and resorption/embryo loss should be considered to evaluate fertility endpoints in Prdx4^−/y^ mice. The testicular treatment of heat stress significantly increased the ROS level and weakened the antioxidant capacity of testicular cells, inducing OS, ERS, and cell apoptosis [42]. Precise sampling time point in heat stress model should be explored more in our future work. We inferred that the effect of Prdx4 KO in young mice could be compensated by other antioxidant enzymes. However, Prdx4 showed a vital antioxidative effect following the strong OS induced by heat stress. Therefore, Prdx4^−/y^ adult mice showed the significant injury in spermatogenesis and testosterone production when caught in OS, suggesting that Prdx4 has powerful potential to ameliorate the OS damage.

In previous studies, Fe^2+^ overload can damage testicular tissue, and the ROS generated by excessive Fe^2+^ is an important source of cell damage [43]. The blockade of SLC7A11 as a key hub in regulating Fe^2+^ overload can induce ferroptosis. xCT‐GPX4 is the main antioxidant pathway [44, 45, 46]. Additionally, it was reported that autophagy‐related proteins are also involved in ferroptosis [47]. In the present study, our results showed that the ablation of Prdx4 led to the Fe^2+^ accumulation in testes under heat stress, increased the lipid peroxidation levels, and decreased the levels of SLC7A11and GPX4 in the Prdx4^−/y^ adult mice. Therefore, the synthesis of GPX4 was reduced, and the balance between oxidative and antioxidant was disordered, resulting in the elevated ferroptosis. In addition, our study proved that the Prdx4 KO Leydig cells mimicked the H_2_O_2_‐induced ferroptosis, leading to mitochondria‐derived OS and apoptosis, which were partially restored by DFO and the overexpressing SLC7A11. Therefore, our findings confirm that Prdx4 functions as an antioxidant by inhibiting ferroptosis in testes.

The development of LOH is facilitated by the process of senescence and exerts adverse impacts on male fertility [48]. In the present study, we found that the level of OS and ERS was significantly increased in the Prdx4 KO aging mice compared with the WT aging mice, accompanied by the significant decline in testosterone level and fertility. In our previous studies, Prdx4 KO accelerated ovarian aging via OS damage and ERS‐related pathways [23]. This study also found the increased ferroptosis in the Prdx4^−/y^ adult mice and testicular Leydig cells under heat stress. Based on which, Prdx4 could be a potential target in inhibiting the accumulation of iron and lipid peroxides and in treating ferroptosis‐associated male reproductive diseases. There has been a natural bioactive triterpenoid extracted from *Tripterygium wilfordii*, which induces ferroptosis in the activated hepatic stellate cells (HSCs) to ameliorate hepatic fibrosis via targeting peroxiredoxins [49]. More work regarding xCT/SLC7A11 activation in the heat‐stressed mice will further validate the mechanism of Prdx4 in vivo. In conclusion, Prdx4 deficiency during aging impairs antioxidant capacity, which cannot be compensated by other antioxidant enzymes, reduces protein folding fidelity, and exacerbates endoplasmic reticulum stress [50]. Prdx4 is capable of ameliorating the decline in testicular function induced by the OS during natural aging, or delaying testicular senescence, indicating that Prdx4 might serve as a potential target for the treatment of LOH. Subsequent research ought to focus on constructing a conditional Prdx4 KO mice, to study the effect of Prdx4 in testicular microenvironment in the future.

## 5 Conclusions

In conclusion, our findings provide a fundamental understanding of Prdx4 in mitigating the adverse effects of the natural senescence and OS. Under severe external challenge, Prdx4 emerges as an indispensable protective factor. These results underscore the essential role of Prdx4 in testicular stress defense, suggesting the potential of Prdx4 as a therapeutic target for the LOH in aging males, and the testicular OS injury in adult men such as non‐obstructive azoospermia, asthenospermia, or teratospermia.

## Author Contributions

Shuning Yuan (Ph.D. student of Prof. Cui and Liu) performed the experiments and wrote the original manuscript. Nini Wei (Master's student of Prof. Cui) participated in most experiments. Shuning Yuan and Nini Wei contributed equally to this manuscript. Weiwei Ma and Mengting Hu (two graduated students of Prof. Cui) performed the preliminary experiment of KO mice. Li Gao and Chao Gao (as two supervising technicians) and Bei Zhang (as a Ph.D. student in Cui's Lab teaching the laboratory techniques) analyzed and visualized the data. Jiayin Liu, Xiaoyu Yang, and Yan Meng participated in project design and discussion, provided critical manuscript review. Yugui Cui (as subject facilitator) designed the project, guided the experiments, and revised the manuscript. All authors approved the manuscript.

## Funding

This study was supported by grants from the National Nature and Science Foundation of China (82171593 and 82271638) and the Innovation Center of Reproductive Medicine in Jiangsu Province of China (CXZX202207). The funding sources were not involved in study design; collection, analysis, and interpretation of data; writing of the report; and the decision to submit the article for publication.

## Ethics Statement

All animal procedures and experiments were performed in accordance with the guidelines for animal care. The design and protocol of animal experiments were approved by the Animal Care Committee of Nanjing Medical University (IACUC‐2307053).

## Conflicts of Interest

The authors declare no conflicts of interest.

## Supporting information




**Supporting File 1**: Supplementary Figure 1 (A) CRISPR/Cas9 system were used to establish the Prdx4^−/y^ mouse model by deleting Prdx4 exon 1–7. To identify the genotype of neonatal mice, PCR genotyping were applied using the following primers for specific amplification of Prdx4: F:5’‐CTGTCTCAAAATAACCACGA‐3’(sense);R:5’‐TAGCTGGATATGTTA GCACT‐3’ (antisense). Genes from wild‐type mice (lanes 1, 3, 6, 8, 10, 12, and 14) and Prdx4^−/y^ mice (lanes 2, 4, 5, 7, 9, and 13) were validated. (B) Schematic of the deleted Prdx4 region.

**Supporting File 2**: Supplementary Figure 2 Ablation of Prdx4 aggravated cytotoxicity in H_2_O_2_‐induced MLTC‐1 cells. (A‐B) Immunoblotting analysis of Prdx4 protein expression in the wild‐type and Prdx4^−/y^ MLTC‐1 cells(n = 3). Vinculin was used as a loading control. (C) Detection of total testosterone using ELISA in MLTC‐1 cells(n = 3). (D) Cell viability of H_2_O_2_‐induced Prdx4^−/y^ MLTC‐1 cells(n = 4). (E) The activity of caspase‐3 in H_2_O_2_‐induced Prdx4^−/y^ MLTC‐1 cells(n = 3). (F‐G) TUNEL staining and the percentage of positive cells in H_2_O_2_‐induced Prdx4^−/y^ MLTC‐1 cells (n = 3). Scale bar = 20 µm. All data are shown as means ± SEMs from at least three independent experiments. Statistical analysis was performed using one‐way ANOVA with Tukey post‐hoc test. ns indicates non‐significant, *p < 0.05, **p < 0.01, ***p < 0.001.

**Supporting File 3**: Andr70221‐sup‐0003‐tableS1.Xlsx

**Supporting File 4**: Supplementary Video. 1 To evaluate sperm quality, the cauda epididymis of every mouse was dissected and minced in HTF at 37°C for 5 min to release and disperse sperm. Then, CASA analysis of wild‐type and Prdx4^−/y^ adult mice were performed. Videos of sperm CASA in WT‐ctrl (Supplementary Video. A), WT‐HS (Supplementary Video. B), Prdx4^−/y^‐ctrl (Supplementary Video. C) and Prdx4^−/y^‐HS (Supplementary Video. D) groups were recorded.

**Supporting File 5**: Andr70221‐sup‐0005‐videoS2.Mp4

**Supporting File 6**: Andr70221‐sup‐0005‐videoS3.Mp4

**Supporting File 7**: Andr70221‐sup‐0005‐videoS4.Mp4

## Data Availability

The data underlying this article will be shared on reasonable request to the corresponding author.
